# Live-Cell, Label-Free Identification of GABAergic and Non-GABAergic Neurons in Primary Cortical Cultures Using Micropatterned Surface

**DOI:** 10.1371/journal.pone.0160987

**Published:** 2016-08-11

**Authors:** Sho Kono, Hideaki Yamamoto, Takatoshi Kushida, Ayumi Hirano-Iwata, Michio Niwano, Takashi Tanii

**Affiliations:** 1 Graduate School of Fundamental Science and Engineering, Waseda University, Tokyo, Japan; 2 Japan Society for the Promotion of Science (JSPS), Tokyo, Japan; 3 Frontier Research Institute for Interdisciplinary Sciences, Tohoku University, Sendai, Japan; 4 Graduate School of Biomedical Engineering, Tohoku University, Sendai, Japan; 5 Research Institute of Electrical Communication, Tohoku University, Sendai, Japan; Universitatsklinikum Wurzburg, GERMANY

## Abstract

Excitatory and inhibitory neurons have distinct roles in cortical dynamics. Here we present a novel method for identifying inhibitory GABAergic neurons from non-GABAergic neurons, which are mostly excitatory glutamatergic neurons, in primary cortical cultures. This was achieved using an asymmetrically designed micropattern that directs an axonal process to the longest pathway. In the current work, we first modified the micropattern geometry to improve cell viability and then studied the axon length from 2 to 7 days *in vitro* (DIV). The cell types of neurons were evaluated retrospectively based on immunoreactivity against GAD67, a marker for inhibitory GABAergic neurons. We found that axons of non-GABAergic neurons grow significantly longer than those of GABAergic neurons in the early stages of development. The optimal threshold for identifying GABAergic and non-GABAergic neurons was evaluated to be 110 μm at 6 DIV. The method does not require any fluorescence labelling and can be carried out on live cells. The accuracy of identification was 98.2%. We confirmed that the high accuracy was due to the use of a micropattern, which standardized the development of cultured neurons. The method promises to be beneficial both for engineering neuronal networks *in vitro* and for basic cellular neuroscience research.

## Introduction

Microfabrication technologies have provided cellular neuroscience with a tool to manipulate cultured neurons at a single cell level, enabling us to engineer living neuronal networks of predetermined structure [1,2]. The major structure of a neuronal network is defined by the type of cells, their connectivity, and direction of the connections. Recent advancements have made it possible, for example, to control axon-dendrite polarity of single neurons [3–6] and to “wire” neurons while they are under culture [7,8]. Even more precise definitions of network structures could be achieved via identification of excitatory-inhibitory cell types. Excitatory neurons release glutamate at synaptic terminals and depolarize postsynaptic cells, whereas inhibitory neurons release γ-aminobutyric acid (GABA) and hyperpolarize postsynaptic cells. These cells have distinct functions in cortical processing [9–11], and hence controlling the cell types in a network is critical in designing neuronal networks *in vitro*.

The most well-established method for identifying the cell types is by immunostaining with specific marker proteins; e.g., alpha-calcium calmodulin-dependent kinase II (α-CAMKII) for excitatory neurons, and GABA or glutamic acid decarboxylase (GAD)-67 for inhibitory neurons [12–14]. This, however, requires chemical fixation of the cells, hence the cell types cannot be identified in live cells. Alternatively, neurons can be transfected with viruses or plasmids to express fluorescent protein under specific promoters [15], but the transfection efficiency is usually <50%, and a high transfection efficiency is inevitably accompanied by cell toxicity. The labelling efficiency issue could be solved by using neurons obtained from transgenic mice that express fluorescent protein in specific cell populations [16,17], but the embedded fluorescence would limit the use of fluorescent dyes in *post hoc* experiments. Under such conditions, it seems appealing to exploit their morphological characteristics during development in culture.

Quite intriguingly, it was found that the temporal difference in excitatory-inhibitory cell types during cortical development is preserved even when neurons were dissociated and brought into culture [12]. Axon formation occurred in the majority of excitatory neurons after 3 days *in vitro* (DIV), whereas most inhibitory neurons did not form axons until 6 DIV. The growth rate of a neurite in a multipolar neuron rapidly increases immediately after it becomes an axon [18]. These findings led us to hypothesize that combining a method to control axon-dendrite polarity using asymmetric micropatterns [6] and monitoring axon growth length on the micropattern would make it possible to identify excitatory and inhibitory neurons, without any labelling or chemical fixation.

The purpose of this work is to improve micropattern design to enhance cell viability at 6 DIV and to show that GABAergic and non-GABAergic neurons can be identified based on their axon length. In the cortex, GABAergic neurons are inhibitory, and the majority of non-GABAergic neurons are excitatory. Micropatterns considered in this study consisted of a circular island for soma adhesion, a single long pathway, and three short pathways. This geometry has been successful in directing axon-dendrite polarity of a single neuron at 2 DIV, with the axon growing on the longest pathway [6]. We first improved the island size and the pathway length so as to culture cortical neurons for a longer term and reveal differences in axon growth. Then axon growth at 2–7 DIV was quantified to find the threshold for axon length for the identification of GABAergic and non-GABAergic neurons.

## Materials and Methods

### Preparation of micropatterned substrates

Micropatterned coverslips were fabricated as described previously [6,8]. Briefly, glass coverslips were cleaned in piranha solution (H_2_SO_4_:H_2_O_2_ = 1:1), and then a 2-[methoxy(polyethyleneoxy)propyl] trimethoxysilane (mPEG) self-assembled monolayer (SAM) was formed by immersing the coverslip overnight in a solution of 5% mPEG in 1% triethylamine-containing toluene under dry N_2_ atmosphere at room temperature. After formation of the SAM, the sample was sonicated in toluene for 10 min and ethanol for 5 min. Next the surface was coated with an electron beam (EB) resist, ZEP-520A (Zeon Chemicals; 60% dilution in ZEP-A solvent), and EB lithography was performed. The developed pattern was transferred to the underlying mPEG SAM by a brief exposure of the sample to O_2_ plasma. The sample was then immersed overnight in a 50 μg/ml poly-D-lysine (PDL)/phosphate-buffered saline solution at room temperature. Finally, the sample was sonicated in tetrahydrofuran and ethanol for 5 min each to remove both unbound PDL and remaining resist. Prior to cell plating, paraffin wax was dotted at corners of coverslips to provide spacing between a glial feeder layer.

### Cell culture

Animal experiments were approved specifically for this study by the Office of Research Ethics, Waseda University (Approval Number: 2013-A102, 2014-A040, 2015-A064, 2016-A069). Timed-pregnant Sprague Dawley rats were obtained from Charles River Laboratories, Japan, and were used immediately after receipt. Primary neurons were obtained from hippocampi or cortices of embryonic day 18 rats and cultured in an N-2 supplemented Minimum Essential Medium in the presence of a glial feeder layer, as described previously [6,19]. Dams and embryos were euthanized by isoflurane exposure and decapitation, respectively. A total of 20 dams were used for experiments.

Cell viability was evaluated based on morphological criteria. For this, cells were imaged under a phase-contrast microscope at indicated time points and were categorized into three groups, i.e., normal, swollen, or ruptured (S1 Fig). Ruptured cells were positive to trypan blue exclusion assay, and hence were biologically dead. Cells with swollen soma were negative to trypan blue staining but are precursors of necrotic death [20,21]; hence the cell would not be suitable as an element in creating neuronal networks. Therefore we used the percentage of normal cells as a criterion for evaluating each pattern. The percentage was calculated by dividing the number of normal cells at indicated time points by total number of cells adhering on micropatterns at 2 DIV. For the sake of readability, we use the term “viability” to refer to the percentage of normal cells. Micropatterns occupied by multiple neurons were excluded from subsequent analyses.

### Immunocytochemistry

The cell types of micropatterned cortical neurons were retrospectively identified by immunofluorescence staining. The cultured neurons were fixed using the method described previously [6]. The following antibodies were used: anti-microtubule associated protein 2 (MAP2) clone HM-2, a mouse monoclonal IgG1 antibody from Sigma diluted 1:1000 to 1:2,000; anti-GAD67 clone 1G10.2, a mouse monoclonal IgG2a antibody from Chemicon diluted 1:1,000; anti-tau-1 clone PC1C6, a mouse monoclonal IgG2a antibody from Chemicon diluted 1:350; Alexa 488-labelled anti-mouse IgG1 antibody from Molecular Probes diluted 1:2,000; Alexa 594-labelled anti-mouse IgG2a antibody from Molecular Probes diluted 1:1,000. The antibodies for MAP2 and tau-1 stain the somatodendritic domain and the axonal process, respectively, of both excitatory and inhibitory neurons. While there is no effective excitatory neuron marker that is available for use in the early stages of culture, GAD67 is a reliable marker for GABAergic neurons, which are major inhibitory cells in the cortex (S2 Fig) [12,15,16].

## Results

### Improving micropattern design to enhance cell viability

**Fig 1:**
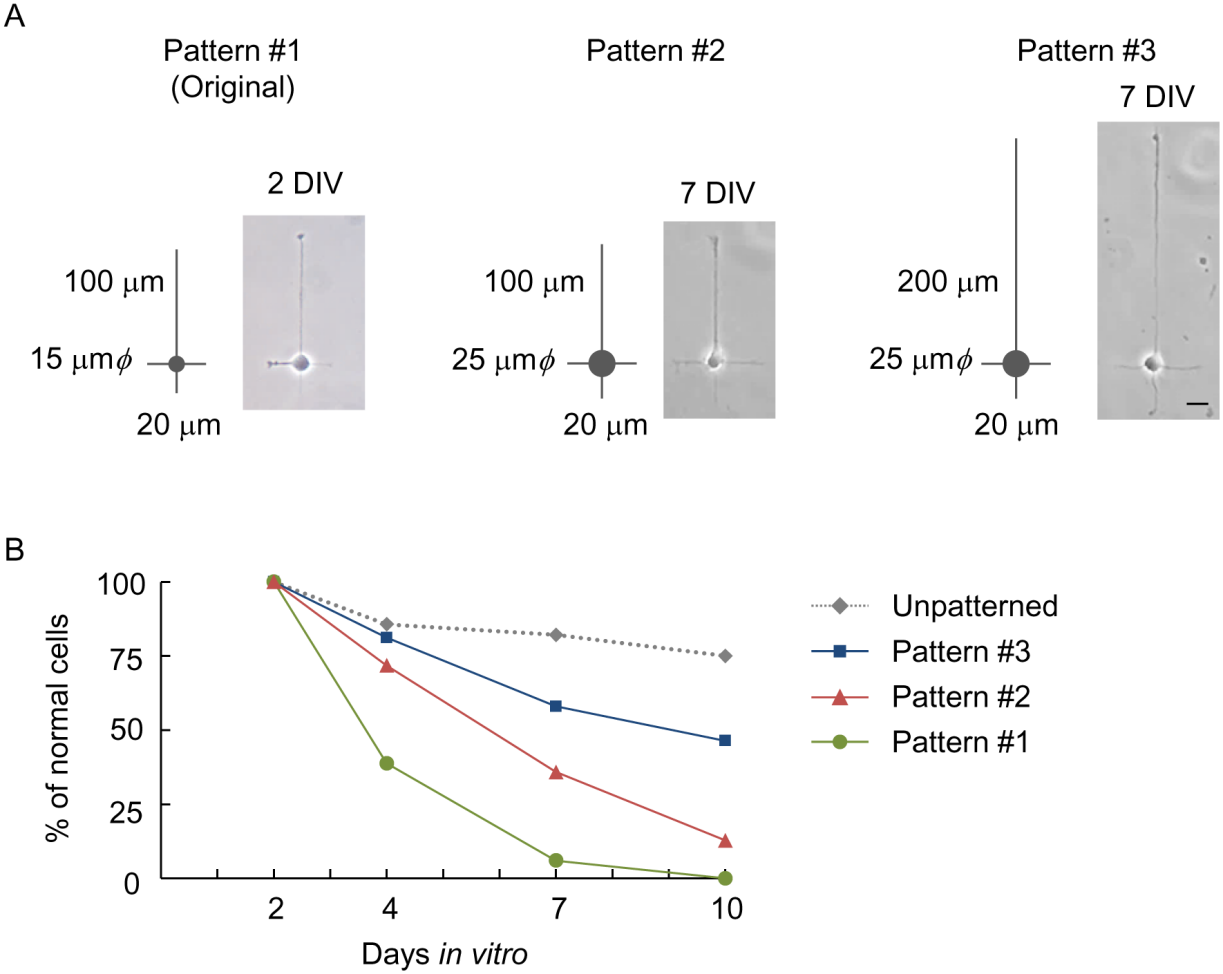
Effect of axon length and soma spreading on viability of micropatterned hippocampal neurons. (A) Neurons on the original (#1) and modified (#2, #3) micropatterns. Pattern #1 consisted of a 15 μm-diameter island for soma adhesion, a single 100 μm pathway for axon growth, and three 20 μm pathways for dendrite growth. Pattern #2 was modified to have a larger (25 μm-diameter) island for soma adhesion. Pattern #3 was further modified to have a larger (25 μm-diameter) island and a longer pathway for axon growth (200 μm). Widths of pathways were 2 μm. (B) Quantified viability in hippocampal cultures on Patterns #1 to #3 and unpatterned coverslips (w/o pattern). The viability of micropatterned neurons was highest on Pattern #3. Scale bar, 20 μm.

The axon-dendrite polarization axis of cultured hippocampal neurons can be controlled via the use of an asymmetric micropattern consisting of a 15 μm-diameter island for soma adhesion, a single 100 μm-pathway for axon growth, and three 20 μm-pathways for dendrite growth (Pattern #1; Fig 1A; 2 DIV) [6]. However, when hippocampal neurons were cultured on the pattern for over 4 DIV, the majority of the neurons became morphologically abnormal and died (Fig 1B). Indeed, the viability at 4 DIV was 39% (*n* = 67 cells), much lower than that on a conventional, unpatterned coverslip (90%; *n* = 56 cells). On the micropattern, the viability further decreased to 6% by 7 DIV, and no cells appeared normal by 10 DIV. Hence we searched for a micropattern geometry that enhances cell viability at later stages of culture.

We extracted the geometrical factors that enhance the viability of hippocampal neurons grown on micropatterns. Hippocampal neurons were used in this experiment, since its cell population is relatively homogenous. As shown in Fig 1B, the viability increased from 6% to 36% at 7 DIV by enlarging the diameter of the circular island for soma adhesion from 15 μm (Pattern #1) to 25 μm (Pattern #2; Fig 1A), and further increased to 58% by lengthening the longest pathway for axon elongation from 100 μm (Pattern #2) to 200 μm (Pattern #3; Fig 1A, S3 Fig). The viability on Pattern #3 was 81% (*n* = 69 cells) at 4 DIV, which was comparable to that on unpatterned coverslips (86%; Fig 1B). A high degree of viability was maintained even at late stages of culture: 58% at 7 DIV and 46% at 10 DIV.

**Fig 2:**
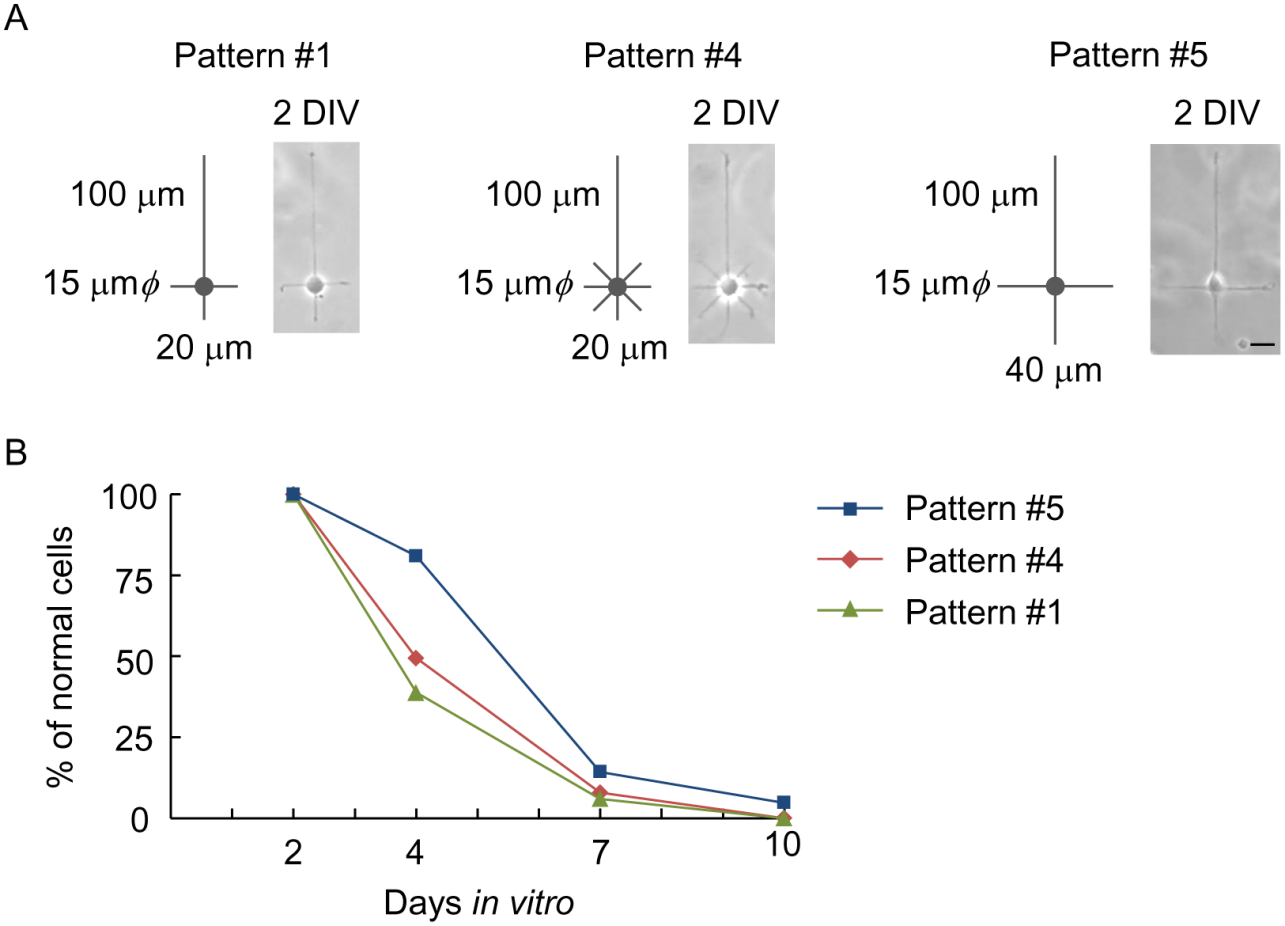
Effect of dendrite geometry on viability of micropatterned hippocampal neurons. (A) Phase-contrast images of neurons cultured on micropatterns with different number and length of short pathways for dendrite elongation (2 DIV). In Pattern #4, the number of short pathways was increased to seven. In Pattern #5, the length was doubled to 40 μm. The island diameter and the length of the longest pathway were fixed to be 15 μm and 100 μm, respectively. (B) Quantified viability of hippocampal neurons on the micropatterns. Scale bar, 20 μm.

Contrary to the substantial effect of axon elongation, modification of the number and length of dendrites had little influence on cell viability (Fig 2). The viability did not increase with increasing the number of short pathways from three to seven (Pattern #4). Although a two-fold increase was observed at 4 DIV by lengthening the short pathways from 20 μm to 40 μm (Pattern #5), the positive effect diminished by 7 DIV. The observations indicate that reducing the degree of soma and axon confinement substantially promotes neuron survival whereas the effect of modifying dendritic geometry is minor.

We also measured the viability of rat cortical neurons on Pattern #3 (*n* = 105 cells). As shown in S4A Fig, the discrepancy in viability between the cortical and hippocampal neurons was negligible, indicating that the results obtained from hippocampal neurons are also applicable to cortical neurons. Immnostaining for an axonal marker revealed that a neurite that grows on the longest pathway differentiates to be its axon (S4B Fig), as expected from the previous study using hippocampal neurons and Pattern #1 [6]. Therefore, we used Pattern #3 in subsequent experiments dealing with the identification of excitatory and inhibitory neurons in cortical cultures.

### Identification of GABAergic and non-GABAergic neurons on micropatterns

**Fig 3:**
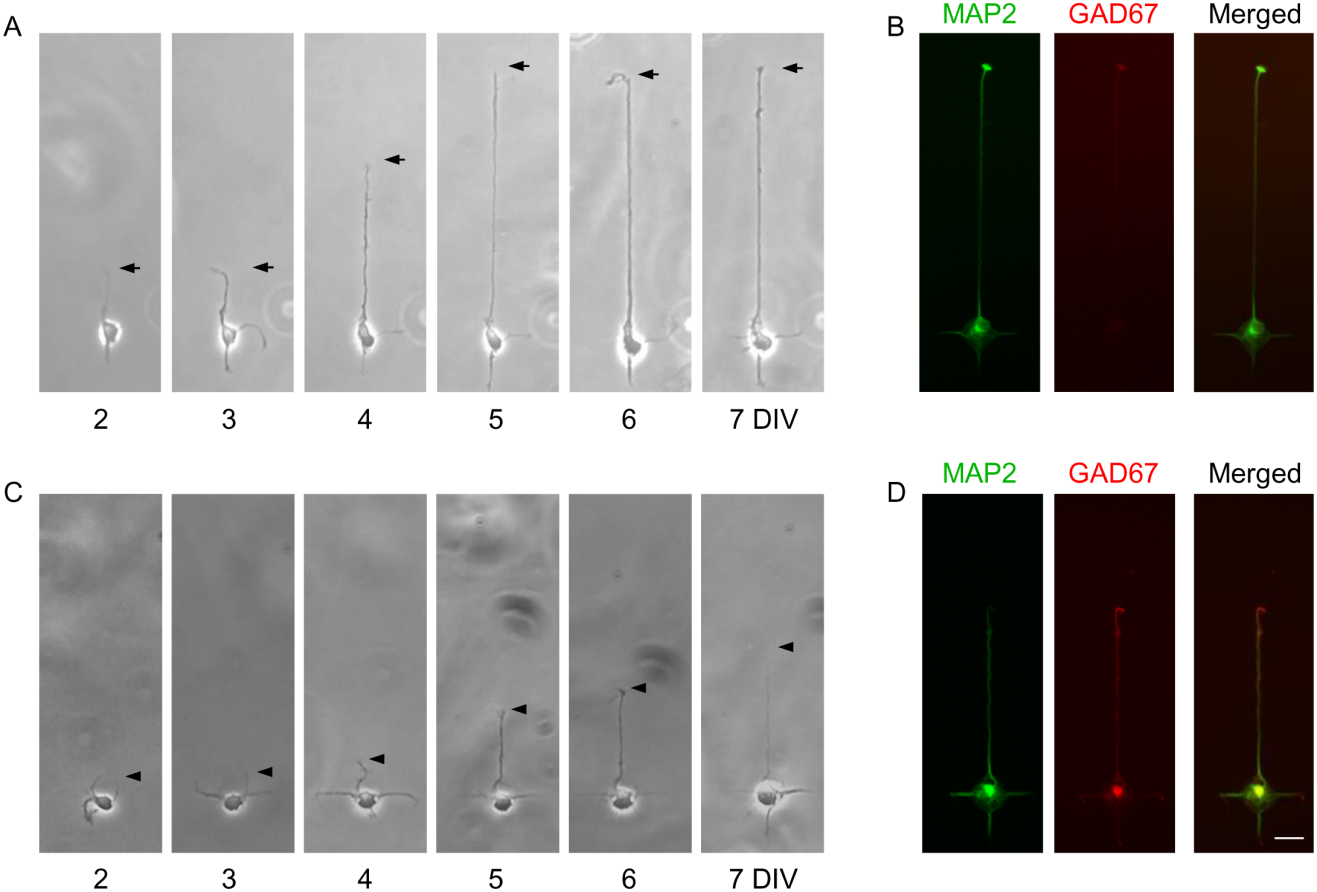
Development of non-GABAergic and GABAergic cortical neurons on micropatterns. Phase-contrast (A,C) and immunofluorescent (B,D) images of rat cortical neurons grown on Pattern #3. Phase-contrast images were taken every day from 2 to 7 DIV, and were fixed and stained at 7 DIV to retrospectively identify the cell type of each neuron (green, MAP2; red, GAD67). Neurons shown in (A) and (B) were non-GABAergic (GAD67−), and those shown in (C) and (D) were GABAergic (GAD67+). By growing neurons on the micropattern, axon is formed in the longest pathway, i.e., in the upward direction. Scale bar, 20 μm.

We next investigated whether excitatory and inhibitory neurons could be identified based on axon length. Cortical neurons consist of both excitatory and inhibitory neurons with a ratio of approximately 4:1 [12]. As shown in Fig 3, the cells were cultured on the improved micropattern (Pattern #3), and the length of a neurite growing on the longest pathway was monitored from 2 to 7 DIV. Their cell types were then determined retrospectively by immunostaining with MAP2 (a marker for both excitatory and inhibitory neurons) and GAD67 (a marker for inhibitory GABAergic neurons). Majority of neurons extended a long process which reached the end of the 200 μm pathway by 5 DIV, and these cells were negative to GAD67 staining (Fig 3B). In contrast, some neurons began to extend long processes at later stage of observations (Fig 3C), and these cells were GAD67+ (Fig 3D).

**Fig 4:**
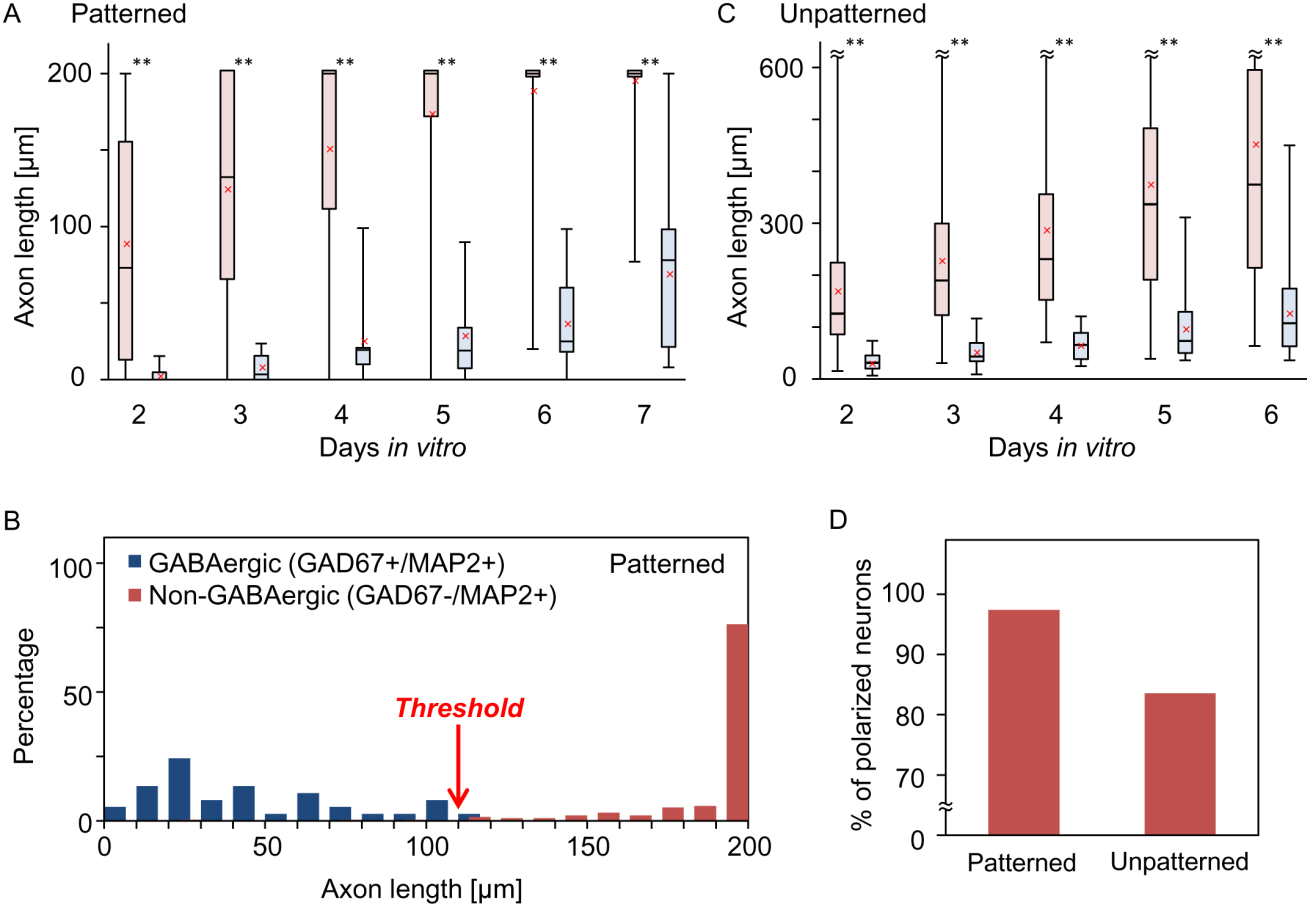
Quantitative comparison of axon growth in non-GABAergic and GABAergic neurons. (A) Box plots of axon lengths of non-GABAergic (red) and GABAergic (blue) neurons on micropatterns (Pattern #3; *n* = 89 cells). The boxes indicate the interquartile range of the data, the range between the 75th percentile and the 25th percentile. Black horizontal lines and red cross-marks indicate the median and average values, respectively. The whiskers represent maximum and minimum values. The micropattern limited the elongation of axons to the length of 200 μm. Asterisks indicate statistical difference between GABAergic and non-GABAergic neurons (** *p* < 0.01). (B) Distribution of axon length at 6 DIV (*n* = 227 cells). The optimal threshold length for distinguishing the two cell types was found to be at 110 μm. (C) Axon lengths of non-GABAergic (red) and GABAergic (blue) neurons cultured on unpatterned surfaces (*n* = 85 cells). (D) Percentage of polarized non-GABAergic neurons at 6 DIV on micropatterns and unpatterned coverslips.

Fig 4A shows the distribution of axon length of GABAergic and non-GABAergic neurons cultured on the micropatterns (*n* = 89 cells; GAD67−:GAD67+ = 76:13). The distribution of axon length measured at the indicated time points clearly shows that axons of non-GABAergic neurons grew faster than that of GABAergic neurons. We determined that the appropriate timing for the identification is at 5 or 6 DIV, because some of neurons did not grow neurites until 4 DIV, and a fraction of the GABAergic neuron axons reached the end of the longest pathway at 7 DIV. In support of this, a statistically significant difference between the two cell types could be observed from 2 to 7 DIV on the micropatterns, with the smallest *p*-value at 6 DIV (*p* < 0.001, Mann-Whitney *U* test). By setting the threshold length at 110 μm at 6 DIV, the two cell types could be distinguished with an accuracy of 98.2% (*n* = 227 cells). A neuron bearing an axon shorter than the threshold was GABAergic, whereas a neuron with a longer axon was non-GABAergic (Fig 4B). The detailed analyses of the statistics are summarized in S1 and S2 Tables.

Next, to investigate the influence of micropatterns on axon growth, we studied the axon length of cortical neurons growing on unpatterned coverslips (Fig 4C; *n* = 85 cells; GAD67−:GAD67+ = 67:18). Similar to the observation on micropatterns, axon of GABAergic neurons grew significantly slower than those of non-GABAergic neurons. However, axon length was more widely distributed than that on micropatterns. The standard deviations of axon length of non-GABAergic and GABAergic neurons cultured on micropatterns were evaluated to be 21 μm and 31 μm at 6 DIV, respectively. Meanwhile, on unpatterned coverslips, the standard deviations of axon lengths of non-GABAergic and GABAergic neurons were determined to be 299 μm and 100 μm, respectively.

Lastly, to clarify the reason that the axon length of neurons cultured on unpatterned coverslips distributes more widely than those on micropatterns, we investigated the timing of axon-dendrite polarization of cortical neurons on unpatterned coverslip and micropatterns. Here we restricted the analysis to non-GABAergic neurons, the majority of which are excitatory glutamatergic neurons. The following criterion for neuron polarization was employed: The length of a neurite becoming an axon is at least 70 μm longer than other neurites. This length criterion for determining neuronal polarization was validated by immunostaining cortical neurons with MAP2 and tau-1 (S5 Fig). We found that 84% of the neurons on unpatterned coverslips (*n* = 67 cells) were polarized by 6 DIV, whereas the percentage on micropatterns was 97% (*n* = 76 cells) (Fig 4D). This implies that micropatterns standardize neuronal development by aligning the length and number of its dendritic processes, thereby clarifying the difference in the timing of axon-dendrite polarization.

## Discussion

In this work, we carried out a comprehensive study of the cause of decreased cell viability of micropatterned hippocampal neurons to find that confinement of axons, but not dendrites, to short lengths and small diameter of the soma adhesion site decreased the viability. Based on the observation, we modified the micropattern geometry that realized 58% and 46% viability at 7 and 10 DIV, respectively. Comparative results were also obtained for cortical neurons. Finally, using the modified micropattern, we quantitatively compared the axon length of GABAergic and non-GABAergic neurons in cortical cultures, and the results showed that the two cell types are distinguishable by setting a threshold axon length of 110 μm at 6 DIV.

Inhibition of cell spreading is known to trigger apoptotic cell death in normal endothelial cells [22,23], although the molecular mechanism for how the geometrical factor is transduced to cell fate is unknown, other than the possible involvement of integrin-mediated signaling through Rho-related proteins, such as Rac GTPase [24]. Interestingly, the morphology of damaged neurons we observed resembled that of cells dying through necrosis—swelling of the cytoplasm at early stage, followed by a rupture of the plasma membrane (S1 Fig) [20,21]. Further molecular studies would clarify the detailed mechanisms underlying the commonalities and differences in various cell types.

The viability of hippocampal neurons on micropatterns increased when the degree of axon and soma confinement was reduced (Pattern #3). To the contrary, the effect of modifying dendritic geometry was minor. In general, cultured hippocampal neurons undergo a very prototypical development, which starts by growing multiple short neurites at 0.5–1.5 DIV [25]. Later, at ~2 DIV, one of the neurites is specified as the axon and it then begins to grow longer than the others [26]. The remaining neurites become dendrites later and continue to grow in length, but their growth rates (~12 μm/day) are much slower than that of axons (~70 μm/day) [25]. Our working hypothesis is that this variation in the intrinsic growth rate of axons and dendrites results in their differential effects on the cell viability.

Neuronal viability on micropatterns, however, was ~25% lower than on an unpatterned substrate, even after optimization. We conjecture that this is mainly due to the limited length of the longest pathway (200 μm). As shown in Fig 4A, most of the axons of the non-GABAergic neurons reached the end of the longest pathway by 7 DIV, and longer growth was then inhibited. Since excessive cell confinement reduces cell viability [23], axon confinement can cause a decrease in viability. Although the viability would be expected to increase by extending the length of the longest pathway, it inevitably comes with a price of lowering the density of micropatterns on a chip, which is an important parameter when designing neuronal circuits with the micropatterned neurons. Hence the 200 μm pattern was used in this study.

The time course for axon formation in excitatory and inhibitory cortical neurons in dissociated culture is known to follow the intrinsic developmental programs of excitatory and inhibitory cortical neurons *in vivo*. Hayashi *et al*. previously showed that at 3 DIV, 84–88% of non-GABAergic (excitatory) neurons had formed axons, whereas the percentage was only 8% for GABAergic (inhibitory) neurons [12]. In the present work, we harnessed this cellular property and demonstrated that this developmental “time lag” can be utilized as a morphological marker for identifying excitatory and inhibitory neurons.

Our results clearly revealed that the micropattern standardizes the timing of neuronal development and suppresses the cell-to-cell variation in the axon length (Fig 4). This was observed in both GABAergic and non-GABAergic neurons and became more prominent at later stages of cell culture. Our working model is that the subcellular distribution of axon-inducing proteins becomes more uniform among different cells, which evens up the timing of axon specification and axon growth rate. One strong molecular candidate involved in this is Shootin-1, a phosphoinositide-3-kinase interacting protein that accelerates neurite outgrowth [27,28]. Neurite outgrowth, in turn, promotes the accumulation of Shootin-1, since neurite extension decreases the gradient of protein concentration and suppresses the diffusion of proteins back to the cell body. We conjecture that the predefined pathway length in our micropattern regulates this positive feedback between neurite outgrowth and Shootin-1 accumulation.

For the nearly 30 years, cultured neurons have been employed in neuroscience to study neuronal development [3–6,12,18,25–30] and synapse functions [17,31,32]. Yet the fundamental difference in network topology between cultured networks and the brain have posed serious issues when using cultured neuronal networks in circuit level research. The gap is now being bridged by the advancement of microfabrication technologies, which enable sophisticated control over cellular connectivity and structure of cultured networks [1,2,7,8,15,33–38]. The findings presented here further extend this approach by manipulating single cells to construct neuronal networks with predetermined neuronal polarity and cell types and will provide a new experimental system to study, for example, structure-function relationships in neuronal networks.

## Supporting Information

S1 Fig Morphological classification of micropatterned neurons. Hippocampal neurons grown on micropatterns were classified as (A) normal, (B) swollen, or (C) ruptured, based on phase-contrast observations. Scale bars, 20 μm.
(TIF)

S2 Fig Distribution of GAD67 immunoreactivity. Cortical neurons were grown on micropatterns (Pattern #3) and were stained with a GAD67 antibody at 7 DIV. The fluorescence intensity was distributed bimodally with the higher and lower peaks corresponding to GABAergic and non-GABAergic neurons, respectively. GABAergic neurons comprised ~16% of the population, in agreement with previous reports [39].
(TIF)

S3 Fig Effect of axon length on the viability of micropatterned neurons. (A) Phase-contrast images of neurons cultured on micropatterns with different length of pathways for axon elongation. The island diameter and short pathway length were kept fixed at 15 μm and 20 μm, respectively. Scale bar, 20 μm. (B) Quantified viability in hippocampal cultures on the micropatterns. The viability at 7 DIV increased by extending the pathway from 200 μm to 400 μm, but the effect was not significant at 10 DIV. Furthermore, negative side effects of pathway elongation, such as the sparseness of micropattern arrays and cell attachment to the pathways, was not negligible on the 400 μm patterns.
(TIF)

S4 Fig Viability and axon-dendrite polarization of cortical neurons on micropatterns. (A) Viability of cortical neurons on Pattern #3 (*n* = 105 cells). The values for hippocampal neurons are plotted as a reference. (B) Double immunostaining for MAP2 (somatodendritic marker, green) and tau-1 (axon marker, red). A cortical neuron was grown on Pattern #3 for 3 days. The neuron has a single tau-1+ neurite (axon) that is oriented upward in the direction of the longest pathway. Scale bar, 50 μm.
(TIF)

S5 Fig Distribution of the neurite length difference (Δ*l*) in unpolarized neurons. Δ*l* is defined as the difference in length between the longest and the second-longest neurites. Cortical neurons were grown on unpatterned coverslips for 2 days and were stained with MAP2 (neuronal marker) and tau-1 (axon marker) (*n* = 25 cells). A neuron was classified as unpolarized if (1) the cell was MAP2+ and (2) the cell did not bear any tau-1+ neurite.
(TIF)

S1 Table True positive/negative, false positive/negative table of the cell-type identification based on axon length. Positive (negative) in axon lengths refers to axons shorter (longer) than 110 μm at 6 DIV.
(TIF)

S2 Table Summary of accuracy, sensitivity and specificity for excitatory neurons, positive predictive value (PPV), negative predictive value (NPV). TP, true positive; TN, true negative; FP, false positive; FN, false negative.
(TIF)
