# Supplementary figures and images for: Live-Cell, Label-Free Identification of GABAergic and Non-GABAergic Neurons in Primary Cortical Cultures Using Micropatterned Surface
Source: PLoS One. 2016 Aug 11;11(8):e0160987. doi: 10.1371/journal.pone.0160987 (PMC4981301; doi:10.1371/journal.pone.0160987)

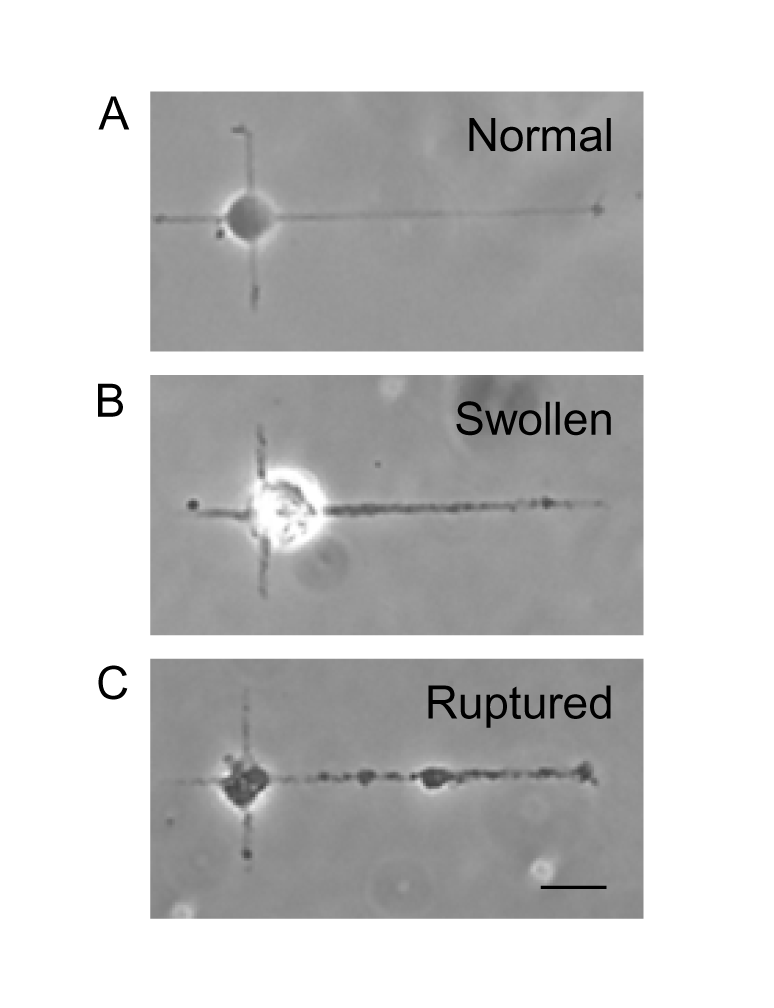

Supplement: S1 Fig — Hippocampal neurons grown on micropatterns were classified as (A) normal, (B) swollen, or (C) ruptured, based on phase-contrast observations. Scale bars, 20 μm. (TIF) [file pone.0160987.s001.tif]

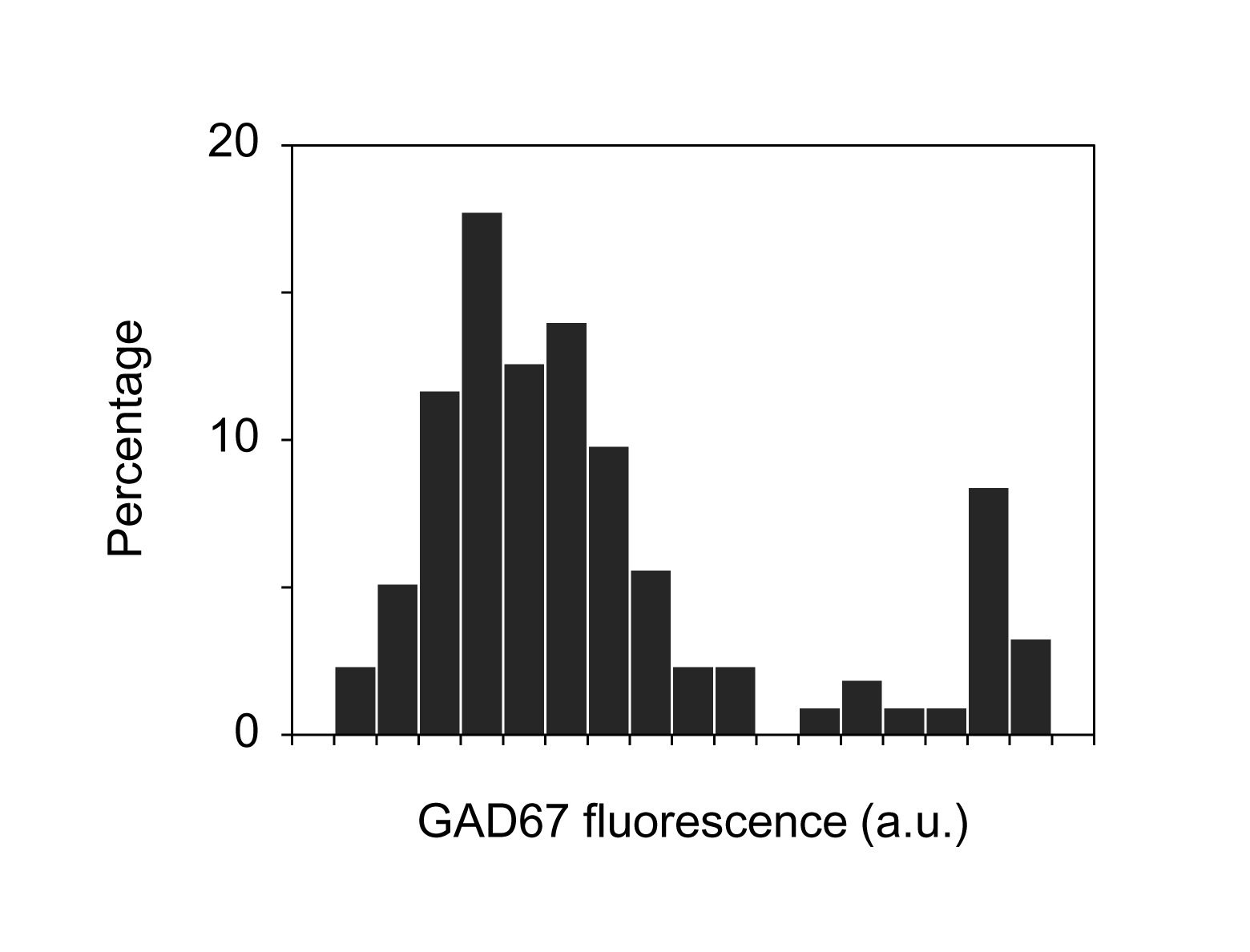

Supplement: S2 Fig — Cortical neurons were grown on micropatterns (Pattern #3) and were stained with a GAD67 antibody at 7 DIV. The fluorescence intensity was distributed bimodally with the higher and lower peaks corresponding to GABAergic and non-GABAergic neurons, respectively. GABAergic neurons comprised ~16% of the population, in agreement with previous reports [39]. (TIF) [file pone.0160987.s002.tif]

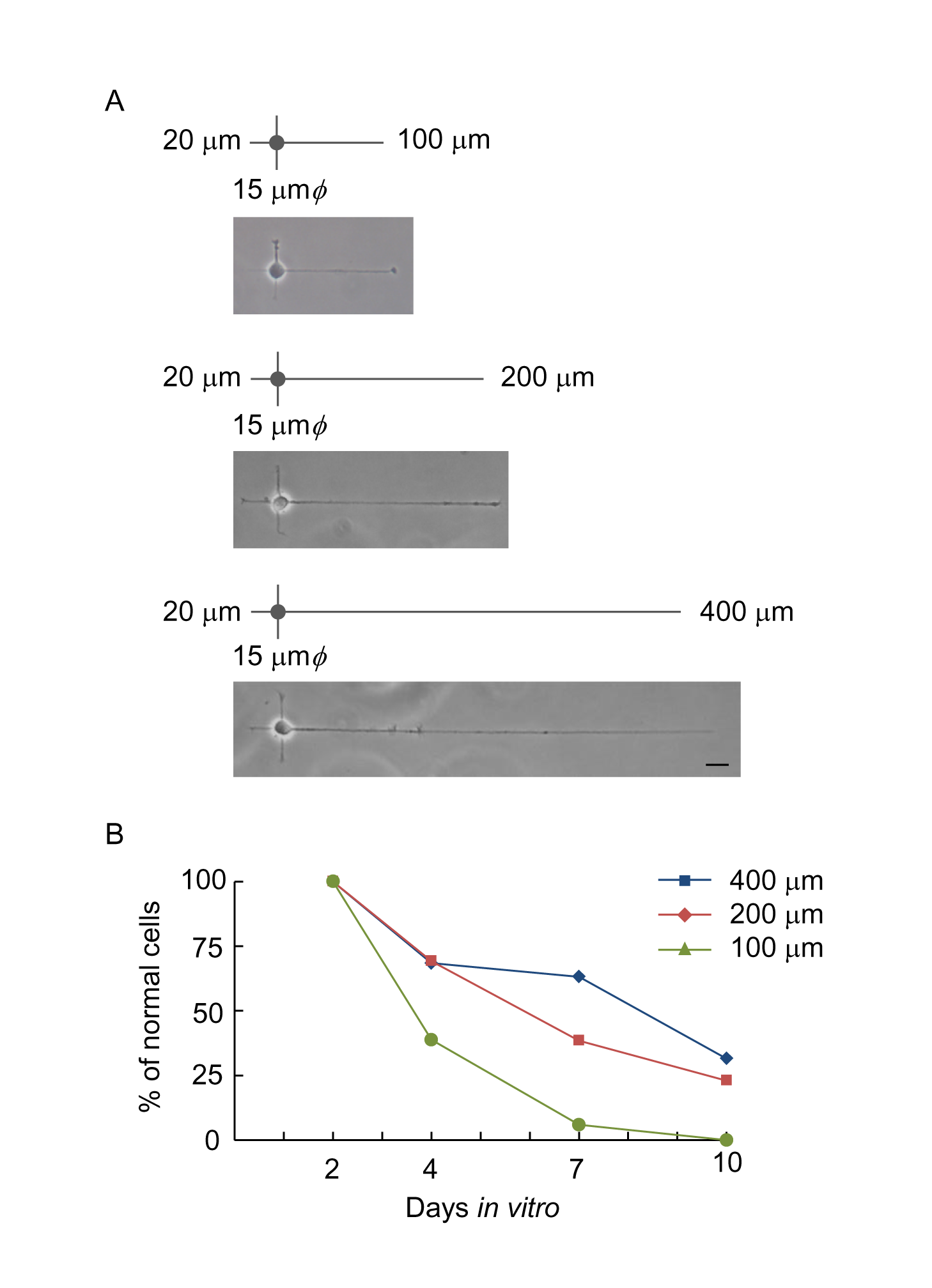

Supplement: S3 Fig — (A) Phase-contrast images of neurons cultured on micropatterns with different length of pathways for axon elongation. The island diameter and short pathway length were kept fixed at 15 μm and 20 μm, respectively. Scale bar, 20 μm. (B) Quantified viability in hippocampal cultures on the micropatterns. The viability at 7 DIV increased by extending the pathway from 200 μm to 400 μm, but the effect was not significant at 10 DIV. Furthermore, negative side effects of pathway elongation, such as the sparseness of micropattern arrays and cell attachment to the pathways, was not negligible on the 400 μm patterns. (TIF) [file pone.0160987.s003.tif]

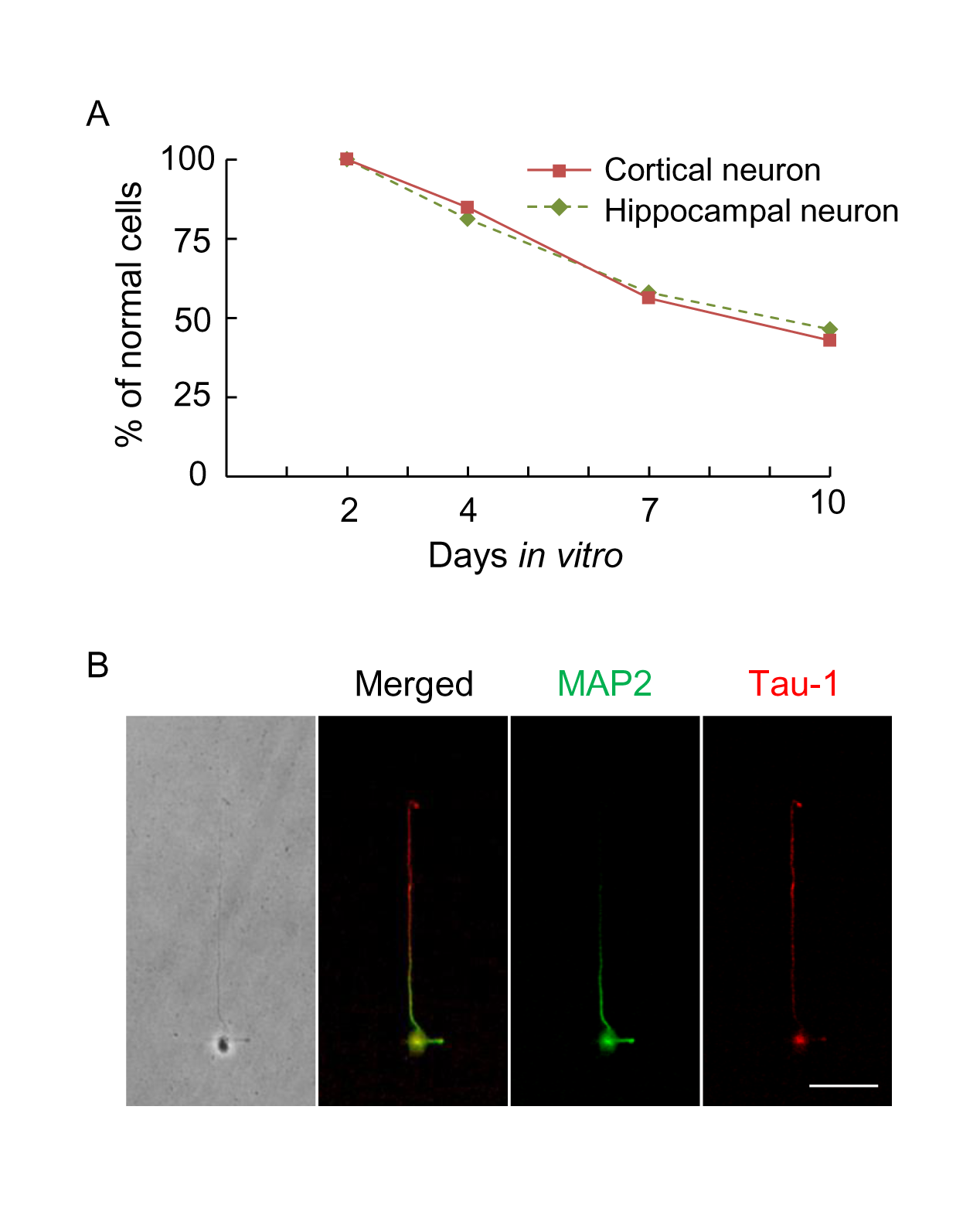

Supplement: S4 Fig — (A) Viability of cortical neurons on Pattern #3 (n = 105 cells). The values for hippocampal neurons are plotted as a reference. (B) Double immunostaining for MAP2 (somatodendritic marker, green) and tau-1 (axon marker, red). A cortical neuron was grown on Pattern #3 for 3 days. The neuron has a single tau-1+ neurite (axon) that is oriented upward in the direction of the longest pathway. Scale bar, 50 μm. (TIF) [file pone.0160987.s004.tif]

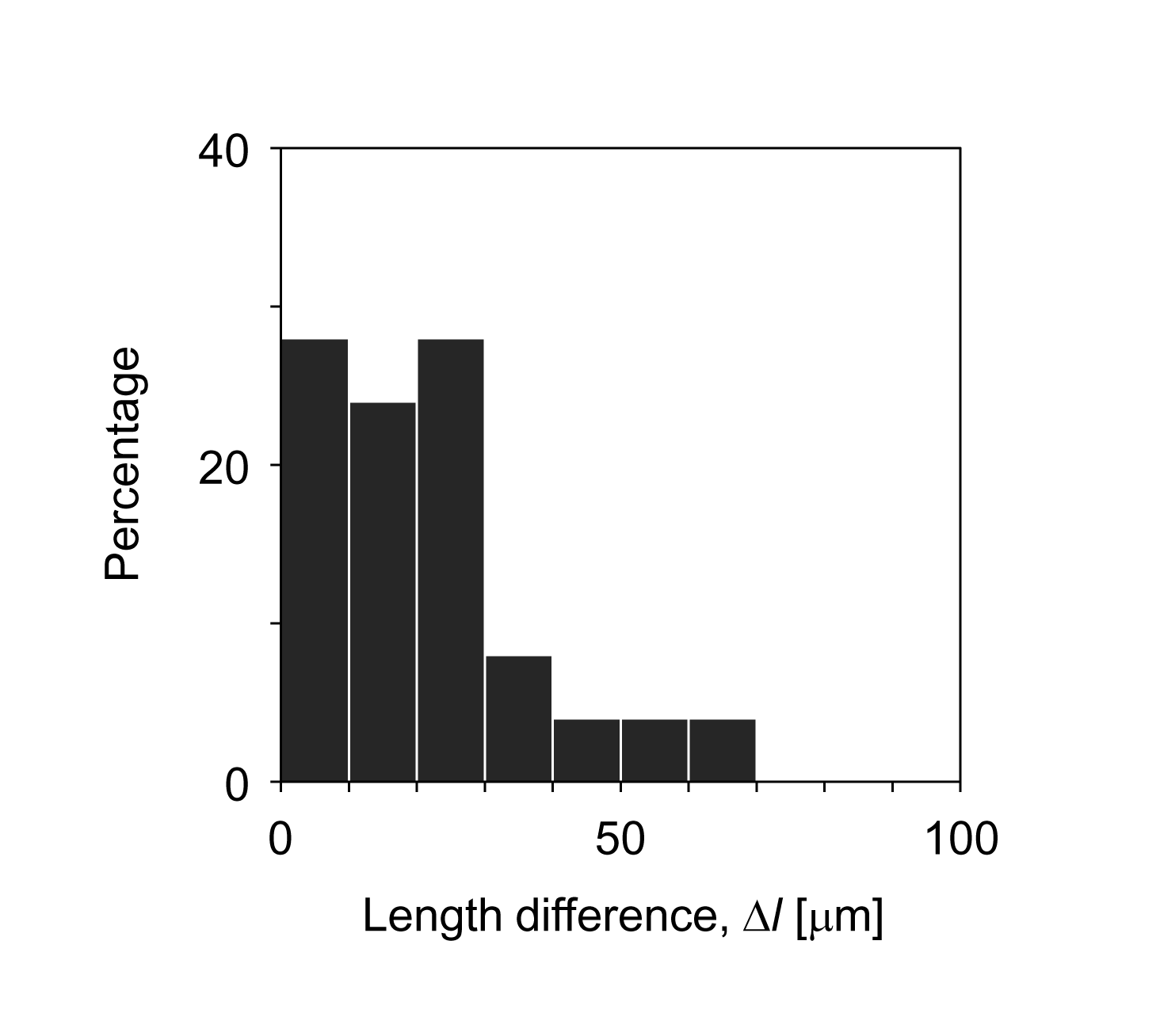

Supplement: S5 Fig — Δl is defined as the difference in length between the longest and the second-longest neurites. Cortical neurons were grown on unpatterned coverslips for 2 days and were stained with MAP2 (neuronal marker) and tau-1 (axon marker) (n = 25 cells). A neuron was classified as unpolarized if (1) the cell was MAP2+ and (2) the cell did not bear any tau-1+ neurite. (TIF) [file pone.0160987.s005.tif]

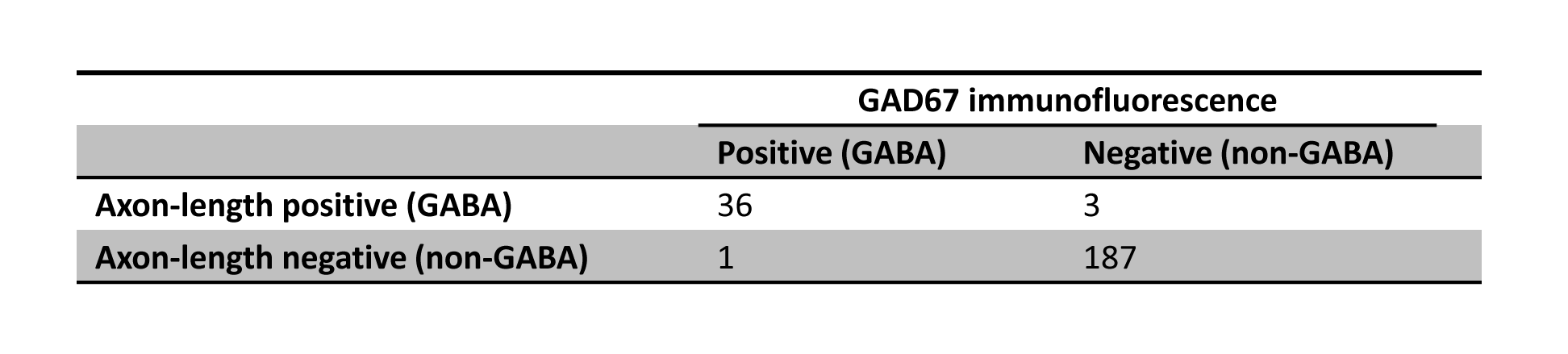

Supplement: S1 Table — Positive (negative) in axon lengths refers to axons shorter (longer) than 110 μm at 6 DIV. (TIF) [file pone.0160987.s006.tif]

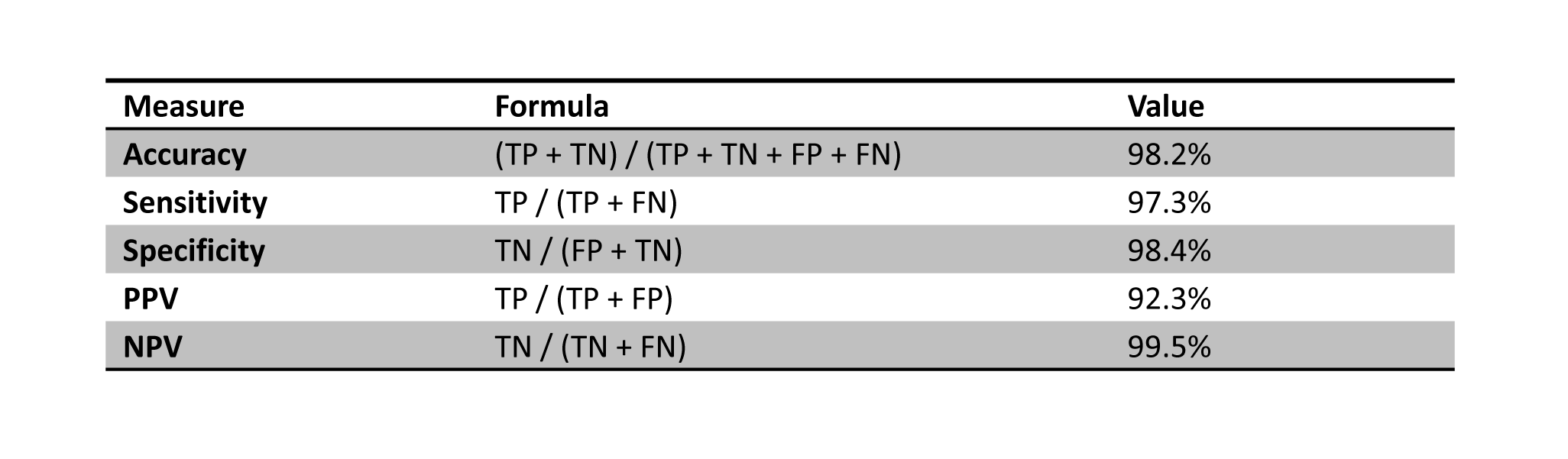

Supplement: S2 Table — TP, true positive; TN, true negative; FP, false positive; FN, false negative. (TIF) [file pone.0160987.s007.tif]
